# Motor proteins at the mitochondria–cytoskeleton interface

**DOI:** 10.1242/jcs.226084

**Published:** 2021-04-15

**Authors:** Antonina J. Kruppa, Folma Buss

**Affiliations:** Cambridge Institute for Medical Research, Department of Clinical Biochemistry, University of Cambridge, Cambridge Biomedical Campus, The Keith Peters Building, Hills Road, Cambridge CB2 0XY, UK

**Keywords:** Myosin, Kinesin, Dynein, Actin, Microtubules, Mitochondria

## Abstract

Mitochondria are multifunctional organelles that not only produce energy for the cell, but are also important for cell signalling, apoptosis and many biosynthetic pathways. In most cell types, they form highly dynamic networks that are constantly remodelled through fission and fusion events, repositioned by motor-dependent transport and degraded when they become dysfunctional. Motor proteins and their tracks are key regulators of mitochondrial homeostasis, and in this Review, we discuss the diverse functions of the three classes of motor proteins associated with mitochondria – the actin-based myosins, as well as the microtubule-based kinesins and dynein. In addition, Miro and TRAK proteins act as adaptors that link kinesin-1 and dynein, as well as myosin of class XIX (MYO19), to mitochondria and coordinate microtubule- and actin-based motor activities. Here, we highlight the roles of motor proteins and motor-linked track dynamics in the transporting and docking of mitochondria, and emphasize their adaptations in specialized cells. Finally, we discuss how motor–cargo complexes mediate changes in mitochondrial morphology through fission and fusion, and how they modulate the turnover of damaged organelles via quality control pathways, such as mitophagy. Understanding the importance of motor proteins for mitochondrial homeostasis will help to elucidate the molecular basis of a number of human diseases.

## Abstract (precis)

**Summary:** This Review highlights the diverse functions of myosin, kinesin and dynein motor proteins in the transport and positioning, fission and fusion, and turnover of mitochondria.

## Introduction

Mitochondria are the powerhouses of the cell, generating adenosine triphosphate (ATP) by oxidative phosphorylation (OXPHOS). In addition to producing energy, they also play critical roles in the biosynthesis of macromolecules, Ca^2^^+^ homeostasis, and signalling in programmed cell death and immunity. Mitochondria are double-membraned organelles of endosymbiotic origin that have an outer mitochondrial membrane (OMM) facing the cytosol and an inner mitochondrial membrane (IMM) marking the boundary of the mitochondrial matrix, which contains the mitochondrial DNA (mtDNA). The intermembrane space (IMS) is sandwiched between the OMM and IMM. Mitochondria can sense stress stimuli, such as nutrient deprivation, and the overall metabolic state of the cell (Chandel, 2015; Giacomello et al., 2020). To maintain organellar homeostasis, mitochondria constantly remodel their network through fission and fusion events, reposition themselves through motor protein- and cytoskeleton-dependent transport, and finally are degraded via several quality control mechanisms (Chan, 2020; Harper et al., 2018; Sleigh et al., 2019). Indeed, defects in mitochondrial homeostasis manifesting as fission–fusion imbalance, impaired transport or reduced clearance of faulty mitochondria by mitophagy increase with age and can lead to a wide range of neurodegenerative disorders (Bakula and Scheibye-Knudsen, 2020; Seo et al., 2010; Sleigh et al., 2019; Tilokani et al., 2018). The overall control of mitochondrial homeostasis involves three types of motor proteins that translocate along cytoskeletal tracks: myosins moving along actin filaments (Box 1), as well as kinesins and dynein moving along microtubules (MTs; Box 2; Vale and Milligan, 2000). The key adaptor complex linking kinesin-1 and dynein to mitochondria consists of Miro1 or Miro2 (referred to collectively as Miro1/2; also known as RHOT1 or RHOT2, respectively) and trafficking kinesin protein 1 or 2 (TRAK1 or TRAK2, referred to collectively as TRAK1/2), and is hereafter referred to as the Miro–TRAK complex (Eberhardt et al., 2020; Melkov and Abdu, 2018). Interestingly, Miro1/2 without TRAK1/2 can also recruit myosin of class XIX (MYO19) to mitochondria (Bocanegra et al., 2020a).

**Box 1. Myosin motors translocate along actin filaments**

Actin monomers polymerize to form actin filaments. A host of actin-binding proteins regulate filament dynamics and the assembly into higher-ordered structures, such as stress fibres, lamellipodia, filopodia and actin networks associated with the plasma membrane or intracellular membranes (Rottner et al., 2017). These include: (1) actin nucleators, such as the ARP2/3 complex, formins and tandem WH2 domain-containing proteins, such as mitoSPIRE; (2) actin filament cross-linking or bundling proteins, such as fascin; (3) actin disassembly factors; and (4) monomer-sequestering proteins.

Myosins are a diverse family of molecular motors that undergo directed movement along actin filaments; they form dynamic tethers between cellular membrane compartments and the actin cytoskeleton. In addition, several classes of myosin motors are actively involved in regulating organization of the actin cytoskeleton (Masters et al., 2016). A total of 39 myosins belonging to 12 classes are expressed in humans. All known myosins, regardless of species, have the same basic structure: an N-terminal motor domain, a lever arm and a tail domain (see figure showing the domain organization of myosin V) (Masters et al., 2016). *In vitro* experiments with myosins indicate that their behaviour broadly falls into three types – transporters, tethers or contractile motors. The lever arm, which typically contains one or more calmodulin- or light-chain-binding IQ motifs, amplifies structural changes in the motor domain into large steps along the actin track. The tail domains either self-assemble to form filaments or contain the majority of binding sites for lipid membranes and cargo adaptor proteins, which recruit the motor to different organelles and vesicular compartments.

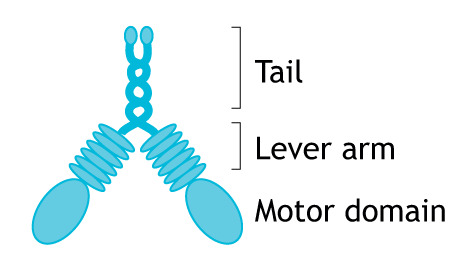

**Box 2. Microtubule-based kinesin and dynein motor proteins**

MTs are rigid but dynamic polymers, growing and shrinking through the addition or loss of α-tubulin–β-tubulin heterodimers, which provide the tracks for polarized transport (Brouhard and Rice, 2018).

Kinesins are a diverse family of motor proteins that transport cargo unidirectionally along MTs (mostly towards the plus end) and regulate MT network organization (Endow et al., 2010). The kinesin superfamily consists of 15 classes, with over 45 known kinesins expressed in mammalian cells. All kinesins share the same basic domain organization: a conserved head domain that is structurally homologous to the myosin motor domain, followed by the neck linker, a divergent stalk, and a tail domain (Hirokawa et al., 2009; see figure, top). Cargo can either bind directly or through adaptor proteins to the kinesin tail or to one of the kinesin light chains that bind to the kinesin heavy chain (Verhey and Hammond, 2009). Kinesins can be grouped according to the position of their motor domain. Kinesins with motor domains at either the N- or C-terminus drive plus- or minus-end directed transport, respectively, whereas kinesins with the motor domain in the middle of the protein are involved in depolymerizing MTs.

Cytoplasmic dynein 1 (referred to as dynein) drives transport of a wide range of cargos towards the minus-end of MTs (Reck-Peterson et al., 2018). Human dynein is a 1.5 MDa complex comprising the dynein heavy chain (DHC, also known as DYNC1H1), the dynein intermediate chain (DIC), the dynein light intermediate chain (DLIC) and three classes of dynein light chains (DLCs; Robl, LC8 and Tctex) (see figure, middle). The DHC consists of an N-terminal tail domain that is important for their dimerization and that binds to DIC and DLIC, and a linker region connecting the tail domain to the C-terminal motor domain, which comprises a ring of six AAA+ domains, a coiled-coil stalk and a MT-binding domain (MTBD). Dynein motility along MTs is powered by ATP hydrolysis in the AAA+ ring; however, processive movement of dynein requires the co-factor dynactin (see figure, bottom). Dynactin is a 1.1 MDa protein complex with 23 subunits assembled around an actin-like filament (ARP1 filament) and p150^glued^ (also known as DCTN1), which can also bind to MTs (Olenick and Holzbaur, 2019). Cargo attachment to dynein requires long coiled-coil activating adaptors, such as BICD, Hook, and Spindly proteins, which bind to the C-terminus of DLIC and run along the dynactin filament (Olenick and Holzbaur, 2019).

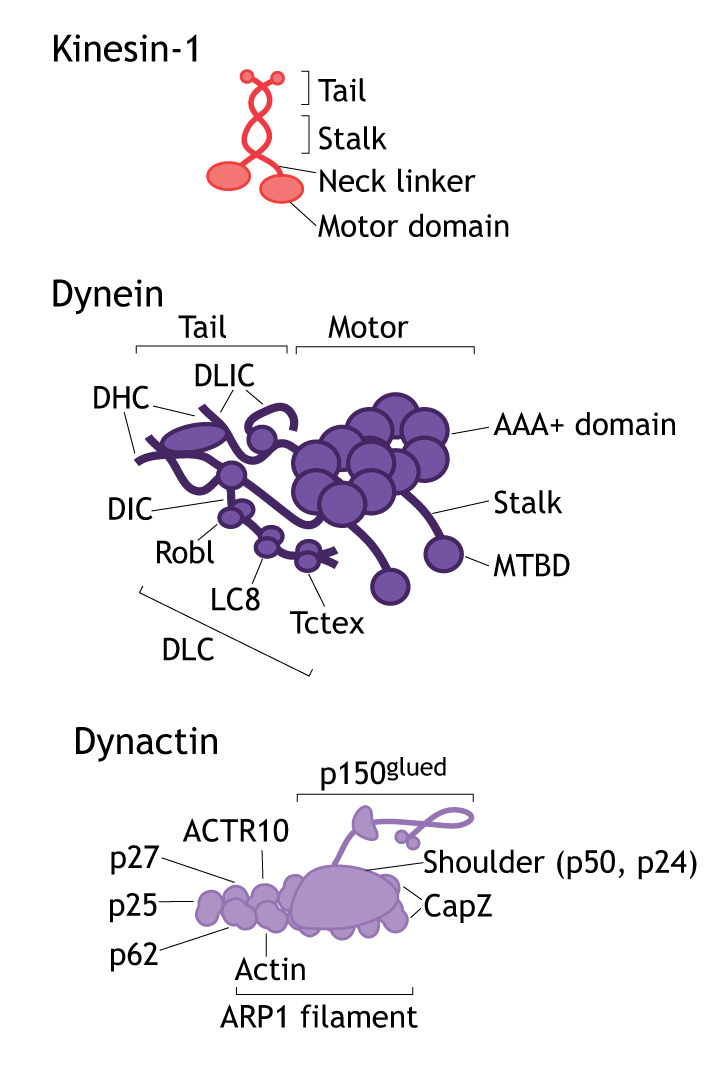

In this Review, we will discuss the importance of motor proteins and motor-linked track dynamics not only for the movement of mitochondria but also for the positioning of these organelles, for the production of force associated with fission and fusion, and for the three-dimensional organization of the cytoskeleton around mitochondria. We will focus on kinesin-1, kinesin-3 and related family members, cytoplasmic dynein-1, MYO19 and myosin V (MYO5), which are involved in mitochondrial transport, as well as non-muscle myosin II (NMII) and myosin VI (MYO6), which have prominent roles in mitochondrial fission and mitophagy, respectively. Finally, we highlight human diseases with mitochondrial phenotypes caused by mutations in motor proteins that open up potential new avenues for treatment (Box 3).

**Box 3. Mutations in motor proteins linked to human diseases**

Motor proteins and other members of the machinery important for mitochondrial dynamics that have been linked to human diseases are summarized in Table S1 (see also table 1 in Tilokani et al., 2018). For instance, a mutation in *MYH14* (encoding the heavy chain of NMIIC) affects mitochondrial fission and leads to a peripheral neuropathy (Almutawa et al., 2019). Fibroblasts from patients harbouring the mutations K671E or I584L in *DYNC1H1*, which have been linked to the neuromuscular disorder spinal muscular atrophy lower extremity-predominant 1 (SMA-LED), display fragmented and fewer mitochondria with decreased MFN1 levels (Eschbach et al., 2013). *KIF5A* mutations causing myoclonus also lead to mitochondrial abnormalities and manifest as atrophy of type I muscle fibres and complex IV deficiency (Duis et al., 2016). Moreover, mutations in the motor adaptor TRAK1 are linked to epileptic encephalopathy early infantile 68; in TRAK1-deficient patient fibroblasts, the mitochondria are irregularly distributed and have reduced motility, decreased membrane potential and a lower respiratory capacity (Barel et al., 2017). Interestingly, Miro1/2 and MYO19 have not been linked to any Mendelian diseases. In many cases where there are disease-causing mutations in motor proteins, it remains to be established whether the motor-linked disruption in mitochondrial homeostasis is also the underlying cause of the disease. Nevertheless, the emerging importance of motor proteins in maintaining mitochondrial homeostasis might open new avenues for treatment of mitochondria-linked diseases, as motor proteins are attractive drug targets.

## Mitochondrial motility in neurons – a balance between transport and docking

**Fig. 1.**
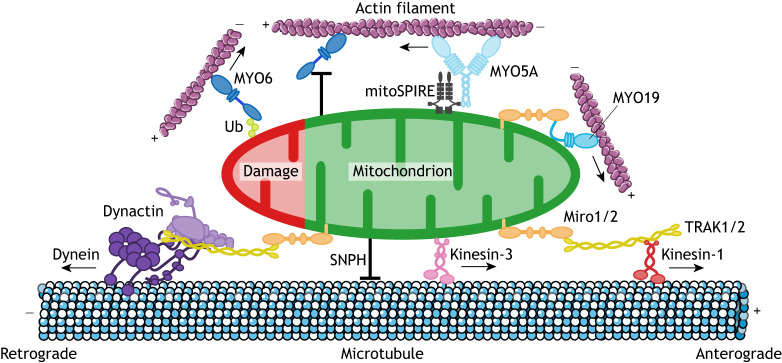
**Mechanisms of motor protein attachment to mitochondria.** Schematic summary of the different classes of myosin, kinesin and dynein motors associated with mitochondria. The MT-based motor proteins, kinesin-1 and the dynein–dynactin complex transport mitochondria towards the plus and minus end of MTs, respectively. They are linked to mitochondria via the adaptor protein TRAK1/2, which binds to the mitochondrially anchored Miro1/2. MYO19 also attaches to mitochondria directly through a lipid-binding region in its tail and by interacting with Miro1/2. Although less well documented than the involvement of kinesin-1 in the movement of mitochondria, kinesin-3 may also play a role in mitochondrial movement towards the plus end of MTs; however, the mechanism of attachment to mitochondria remains unknown. MYO5A is targeted to mitochondria by the mitochondrial isoform of the actin nucleator SPIRE (mitoSPIRE). Syntaphilin (SNPH) is attached to the OMM and acts as an anchor by either directly binding to MTs or to actin filaments via its interaction with MYO6, resulting in stationary mitochondria. During PINK1- and PRKN-mediated mitophagy, MYO6 is recruited to damaged mitochondria by ubiquitin (Ub). Actin filament and microtubule images are adapted from Servier Medical Art (https://smart.servier.com/) under the terms of a CC-BY 3.0 license.

The dynamic positioning of mitochondria within different cell types and tissues is spatially regulated to fulfil subcellular requirements for local energy production and Ca^2+^ signalling, which is particularly important in neurons due to their extended morphology (Seager et al., 2020). Mitochondrial movement over long distances in axons or dendrites is dependent on kinesin and dynein motors moving in opposite directions along MTs due to the intrinsic track polarity. Fast axonal transport with velocities between 0.25 and 1 µm/s is driven in the anterograde direction from the cell body to the distal part of the axon and the synapse by plus-end-directed kinesins, whereas retrograde movement depends on the minus-end-directed dynein motor moving back towards the soma (cell body) (Pilling et al., 2006). This bidirectional transport of mitochondria along axonal MTs, which characteristically displays frequent pauses and directional changes, and the docking mechanisms that ensure the correct distribution of mitochondria throughout the neuron, is fundamentally the same in all eukaryotic cells as it uses equivalent motors and machinery. In contrast to the uniform MT polarity in the axon, neuronal dendrites contain MTs of mixed polarity with approximately equal numbers of plus- and minus-ends of MTs directed towards the cell surface; hence, the track orientation determines the direction of transport and the motor involved (Satoh et al., 2008; van Spronsen et al., 2013; Yan et al., 2013). Mitochondrial transport has been extensively studied in neurons (for examples, see recent reviews by Cardanho-Ramos et al., 2020; Mandal and Drerup, 2019), and we will focus here only on the key motors and mitochondrial adaptors (Fig. 1).

### Kinesins mediate anterograde mitochondrial transport

Kinesin-1 (which in mammals has three heavy chain isoforms – the ubiquitously expressed KIF5B, and neuron-specific KIF5A and KIF5C) is the primary motor for anterograde mitochondrial transport (Fig. 1) in axons in *Drosophila*, mouse and zebrafish (Campbell et al., 2014; Hurd and Saxton, 1996; Karle et al., 2012; Pilling et al., 2006; Tanaka et al., 1998). Kinesin-1 is a highly processive motor that takes over 100 steps before detaching from the MT track *in vitro* (Taylor and Borisy, 2000). Within the cell, kinesin-1 is activated and the run length increases upon binding to TRAK1, thereby promoting kinesin-1-dependent long-range transport of mitochondria in the crowded cytosol (Henrichs et al., 2020).

In contrast to the well-documented role of kinesin-1, the importance of other classes of kinesins for mitochondrial transport remains controversial. The highly processive kinesin-3 protein KIF1B has been suggested to transport mitochondria *in vitro* (Nangaku et al., 1994), although no defects in mitochondrial transport are observed in KIF1B-mutant mice (Zhao et al., 2001) or zebrafish (Drerup et al., 2016; Lyons et al., 2009). Furthermore, one publication has described changes in mitochondrial morphology that are linked to the depletion or overexpression of the dominant-negative tail domain of kinesin-like protein 6 (KLP6, also known as KIF28P), a distantly related member of the kinesin-3 family (Tanaka et al., 2011). Although expression of mutant KLP6 also affects mitochondrial distribution in neurites (Tanaka et al., 2011), further work is required to establish the role of KLP6 in mitochondrial transport. Finally, loss of KIF1-binding protein (KIFBP, also known as KBP and KIF1BP), the adaptor protein for both kinesin-3 and KLP6, has been reported to change mitochondrial distribution in some specific cell types and developmental stages (Drerup et al., 2016; Lyons et al., 2008; Wozniak et al., 2005). Since KIFBP alters MT dynamics and mitochondrial biogenesis, it is more likely to play an indirect role in mitochondrial transport by modulating MT tracks (Atherton et al., 2020; Donato et al., 2017; Kevenaar et al., 2016).

### Dynein transports mitochondria in the retrograde direction

Dynein is the main motor driving the retrograde transport of axonal mitochondria (Fig. 1) (Drerup et al., 2017). Interestingly, this activity also requires kinesin-1, which delivers newly synthesized dynein from the soma via anterograde slow axonal transport out to the synapse (Twelvetrees et al., 2016). The majority of disease-associated dynein mutations affect overall retrograde dynein-dependent motility, whereas mutations causing loss of function in the dynactin subunit ACTR10 (Box 2) selectively impair binding of dynein to mitochondria in zebrafish (Drerup et al., 2017).

### Motor adaptors for mitochondria

Microtubule and myosin motors are bound to the mitochondrial surface by the conserved Miro–TRAK adaptor complex (Fig. 1), which is the key regulator of axonal transport of mitochondria in many different organisms (Schwarz, 2013). TRAK1 and TRAK2, the mammalian homologues of the *Drosophila* Milton protein, bind directly to Miro proteins, which are anchored to the OMM via a C-terminal transmembrane domain (Fransson et al., 2006; Glater et al., 2006). In mammals, two Miro proteins are expressed, Miro1 and Miro2, and both have two GTPase domains flanking two Ca^2+^-sensing EF-hand domains (Fransson et al., 2003; Klosowiak et al., 2013; Macaskill et al., 2009). The TRAK family has an extensive N-terminal coiled-coil region similar to huntingtin-associated protein (HAP1) and a C-terminal domain that binds to Miro proteins (Stowers et al., 2002). Whereas TRAK1 predominantly mediates axonal transport by simultaneously interacting with both the cargo-binding tails of kinesin-1 and dynein (Glater et al., 2006; Smith et al., 1995), TRAK2 promotes dendritic transport by forming a complex with dynein in a conformation that interferes with kinesin-1 binding (Brickley and Stephenson, 2011; Loss and Stephenson, 2015; van Spronsen et al., 2013).

Interestingly, Miro proteins not only regulate transport, but also play a role in maintaining mitochondrial morphology and the architecture of the IMM, since Miro1/2 double-knockout (KO) mouse embryonic fibroblasts (MEFs) have a non-uniform arrangement of mitochondrial cristae (Modi et al., 2019).

Finally, in addition to mitochondrial motility, kinesin-1, together with TRAK1/2, also drives the tubulation of mitochondria in the cell periphery. This process requires kinesin-1 motor activity and gives rise to highly dynamic thin tubules that fuse to form lattices and eventually generate an interconnected mitochondrial network (Wang et al., 2015). Indeed, overexpression of TRAK1/2 leads to striking changes in mitochondrial network morphology, inducing the formation of extended, highly dynamic mitochondrial tubules in a MT-dependent manner (Fransson et al., 2006; Koutsopoulos et al., 2010).

### Docking and anchoring

For mitochondria to become stationary, their movement is either simply paused (Chung et al., 2016; Wang and Schwarz, 2009) or they are actively anchored to either the actin or MT track. Mitochondrial positioning and docking at locations of nutrient abundance increases the efficiency of ATP generation and is caused by stalling due to TRAK1 glycosylation (O-GlcNAcylation) at high glucose levels (Pekkurnaz et al., 2014). Miro proteins can also act as Ca^2+^ sensors and induce mitochondrial immobilization by a Ca^2+^-dependent mechanism, for example, at synaptic sites. Ca^2+^ binding to the EF-hand motifs of Miro1/2 triggers conformational changes that arrest mitochondrial movement by disrupting interactions between kinesin-1 and MTs, or by leading to dissociation of the Miro–TRAK complex from kinesin-1 (Macaskill et al., 2009; Saotome et al., 2008; Wang and Schwarz, 2009). Elevated Ca^2+^ levels also activate anchoring proteins that mediate docking of mitochondria, such as syntaphilin (SNPH). Syntaphilin, previously thought to be axonal-specific, is inserted into the OMM with its C-terminal transmembrane domain and directly binds to MTs via its N-terminal MT-binding domain (Kang et al., 2008). It functions as a brake to axonal mitochondrial transport by competing with TRAK1/2 for kinesin-1 binding and, once bound, inhibiting kinesin-1 motor activity (Chen and Sheng, 2013). The dynein light chain (DLC) LC8 (also known as DYNLL1) is thought to enhance SNPH-based anchoring of mitochondria (Chen et al., 2009b).

Mitochondrial positioning and immobilization may require the inactivation of kinesin and dynein motors, but also involves actin-based myosin motor proteins, which may act as short-range transporters or may regulate mitochondrial tethering. Disrupting the actin cytoskeleton with drug treatments has been shown to reduce mitochondrial anchoring in axons (Gutnick et al., 2019), and disruption of the actin- or MT-based cytoskeleton destabilizes mitochondrial tethering in dendrites (Rangaraju et al., 2019). MYO6 (also known as Jaguar in *Drosophila*) is thought to oppose MT-based mitochondrial movement to facilitate organelle docking in *Drosophila* neurons, in line with recent findings that the cargo-binding domain of MYO6 directly binds to SNPH (Fig. 1) (Li et al., 2020; Pathak et al., 2010). MYO6 is a unique myosin that moves towards the minus ends of actin filaments, in the opposite direction to all other known myosins (Wells et al., 1999). MYO6 has essential roles in membrane trafficking pathways and mediates the delivery of endosomal membranes to autophagosomes during autophagosome maturation (de Jonge et al., 2019). The direct interaction between MYO6 and SNPH suggests a new role for MYO6 in capturing mitochondria on F-actin and mediating the cytoskeletal switch from MTs to actin, which serves as a platform for anchoring organelles in presynaptic terminals (Li et al., 2020). MYO6-dependent mitochondrial capture is regulated by AMP-activated protein kinase (AMPK)–p21-activated kinase (PAK) signalling pathways and phosphorylation of MYO6 in the motor domain (Li et al., 2020).

A role for MYO5 in mitochondrial docking was observed in *Drosophila*, where depletion of MYO5 (encoded by *didum* in *Drosophila*) causes an increase in anterograde and retrograde axonal mitochondrial motility (Pathak et al., 2010). Myosins of class V (MYO5A, -B and -C) function as a dimer and move processively along actin filaments. Each MYO5 heavy chain binds six calmodulins, giving rise to a long lever arm, which allows this myosin to take large steps of 36 nm and move straight along the helical actin track (Mehta et al., 1999). MYO5A can bind to a mitochondrially-anchored isoform of the actin nucleator and tandem WH2 domain-containing protein SPIRE1 (mitoSPIRE), which cooperates with formins to assemble actin filaments (Fig. 1) (Straub et al., 2020 preprint). These actin–myosin networks are thought to oppose mitochondrial motility by anchoring mitochondria, since loss of mitoSPIRE in MEFs increases mitochondrial motility (Straub et al., 2020 preprint).

In vertebrates, MYO19 is tightly associated with mitochondria and regulates the cellular distribution of this organelle (Fig. 1). Overexpression of full-length MYO19 significantly increases the overall mitochondrial motility over short distances in epithelial cells (Quintero et al., 2009). In neuronal cells, however, MYO19 causes a decrease in mitochondrial movement by inducing track switching from MTs to actin filaments, which results in reduced mitochondrial run length (Quintero et al., 2009). MYO19 displays a slow rate of ADP release linked to a prolonged actin-binding state and high duty ratio *in vitro* (Lu et al., 2014; Ušaj and Henn, 2017). The very short unique C-terminal tail of MYO19 contains a lipid-binding region of 30–45 amino acids essential for mitochondrial targeting (Hawthorne et al., 2016; Shneyer et al., 2016). In addition, its far C-terminal tail directly interacts with Miro1/2 and competes with TRAK1/2 for binding (Oeding et al., 2018). Thus, Miro proteins not only form a complex with MT motors but also with MYO19, thereby coordinating MT- and actin-based mitochondrial movement. This is confirmed by studies on fibroblasts isolated from Miro1/2 double-KO mice, which not only display reduced retrograde and anterograde mitochondrial movement along MTs, but have also lost MYO19 from mitochondria (López-Domenéch et al., 2018). The N-terminal GTPase domain of Miro1/2 is critical for recruiting and stabilizing MYO19 on the OMM by facilitating its membrane insertion and protecting it from proteasome-mediated degradation (Bocanegra et al., 2020b; López-Domenéch et al., 2018; Oeding et al., 2018). Finally, MYO19 is also able to regulate the precise localization of mitochondria in response to metabolic stimuli, such as glucose starvation or reactive oxygen species (ROS), which activate MYO19 and lead to re-localization of mitochondria into the tips of filopodia (Shneyer et al., 2016, 2017).

As discussed above, the correct distribution of mitochondria is particularly important in highly polarized neuronal cells. In the next section, we highlight other specialized adaptations regulating mitochondrial positioning.

## Specialized cell adaptations for mitochondrial positioning

### Cardiac cells

Mitochondrial transport in cardiac myoblasts is driven by the same players (kinesin-1, dynein, TRAK1/2) as in neuronal cells and is regulated by similar Ca^2+^-dependent mechanisms. Indeed, Ca^2+^ is a key regulator of contractile activity, and elevated cytoplasmic Ca^2+^ resulting from ionomycin or thapsigargin treatment inhibits mitochondrial transport (Iqbal and Hood, 2014; Yi et al., 2004).

Kinesin-1-mediated mitochondrial transport is also important during cardiac maintenance. Cardiac hypertrophy (cardiomyocyte growth) is an adaptive response aimed at reducing wall stress and maintaining cardiac function (Nakamura and Sadoshima, 2018). Kinesin-1 expression is upregulated in a mouse model of pathological cardiac hypertrophy and in neonatal rat ventricular cardiomyocytes. In these cardiomyocytes, mitochondria are re-localized to the cell periphery accompanied by increased mitochondrial respiration associated with the hypertrophic response, which can be reverted by depletion of kinesin-1 (Tigchelaar et al., 2016). Interestingly, altered mitochondrial morphology and distribution are hallmarks of heart failure (Chen et al., 2009a; Schaper et al., 1991).

The intercellular communication between cardiomyocytes and cardiac myofibroblasts is important for maintaining normal myocardial function and is facilitated by membrane nanotubes (MNTs), which are long, thin membrane-based connections. These connections enable kinesin-1-dependent transport of mitochondria from cell to cell, thus acting as direct highways for the exchange of mitochondria (He et al., 2011; Rustom et al., 2004; Shen et al., 2018; Zhang and Zhang, 2013). Kinesin-1-mediated transport of mitochondria along MTs in MNTs is physiologically important in ischaemic cardiomyopathy for rescuing cardiomyocytes from hypoxia–reoxygenation-induced apoptosis (Shen et al., 2018).

### Migrating cells

Migrating cells have a radial MT organization with plus ends facing the cell periphery (Bettencourt-Dias and Glover, 2007). In migrating lymphocytes, the centrosome, the main MT-organizing centre (MTOC), is located in the uropod at the rear or trailing end of the cell, whereas in motile epithelial cells, the centrosome is oriented towards the migration axis, establishing a high-density MT network towards the leading edge (Barker et al., 2016; Melkov and Abdu, 2018).

In lymphocytes or T cells, mitochondria accumulate in the energy-demanding uropod, where they provide ATP for actomyosin II-dependent force generation required for cell movement (Campello et al., 2006; Sanchez-Madrid and del Pozo, 1999). The Miro1–dynein complex is important in controlling lymphocyte and T cell migration by regulating mitochondrial accumulation around the MTOC at the uropod (Morlino et al., 2014). Another example of polarized distribution of mitochondria can be observed in immune cells during the formation of the immunological synapse between T cells and antigen-presenting cells. Mitochondria regulate sustained Ca^2+^ influx, and kinesin-1 drives anterograde mitochondrial transport to the immunological synapse at the plasma membrane (Quintana et al., 2006, 2007). Miro proteins also regulate mitochondrial recruitment in leukocytes to sites of contact with inflamed endothelial cells (Morlino et al., 2014).

In cancer cells, mitochondrial positioning is crucial for facilitating the energy-expensive processes of dissemination and invasion (da Silva et al., 2014). Mitochondria provide localized energy production by infiltrating the leading edge lamellipodia of migrating epithelial cancer cells in a MT-dependent manner (Cunniff et al., 2013, 2016; McKenzie et al., 2011). Anterior localization of mitochondria correlates with faster cell movement and increased directional persistence, which may account for the ability to invade further and metastasize earlier. Kinesin-1 and Miro1/2 are responsible for transporting mitochondria to the leading edge, since their depletion suppresses mitochondrial re-localization and results in slower cell migration rates as well as reduced tumour cell invasion (Caino et al., 2016; Desai et al., 2013). These findings are supported by observations in Miro1 KO MEFs, which are slower in collective and single-cell migration assays as a result of impaired membrane ruffling, leading-edge protrusion and focal adhesion dynamics (Schuler et al., 2017). Interestingly, downregulation or loss of the SNPH anchor enhances tumour progression in humans, whereas SNPH expression in tumour cells inhibits mitochondrial transport and blocks metastasis *in vivo* (Caino et al., 2016).

### Gametes – oocytes and spermatids

Kinesins and their mitochondrial adaptor proteins are also involved in the spatial redistribution of mitochondria during oocyte maturation and spermatogenesis. Mitochondria in many species (human, *Xenopus* and *Drosophila*) enter oocytes from interconnected germ cells to generate the Balbiani body, a non-membrane-bound compartment packed with mitochondria. In *Drosophila*, oocyte acquisition of mitochondria depends on kinesin-1- and Milton-dependent movement, since mutations in these proteins cause premature and excessive mitochondrial transport into the oocyte (Cox and Spradling, 2003, 2006). During *Drosophila* oogenesis, the long isoform of the Oskar protein tethers mitochondria to the posterior cortex at the site of primordial germ cell formation through an actin-dependent mechanism requiring the non-muscle tropomyosin, Tropomyosin II; however, at present, it is not known whether any myosin motors are involved in this process (Hurd et al., 2016). During meiotic maturation of mouse oocytes, kinesin-3 is responsible for mitochondrial redistribution near the metaphase plate (Kong et al., 2016).

In *Drosophila* spermatogenesis, mitochondria undergo dramatic rearrangements, during which they aggregate and fuse to form the spherical Nebenkern (Varuzhanyan and Chan, 2020). Milton has been implicated in anchoring mitochondria of the Nebenkern at the minus end of MTs to the nucleus (Aldridge et al., 2007). Furthermore, in Milton-mutant flies, the unfurling and elongation of mitochondria along MTs is impaired during axonemal growth (Aldridge et al., 2007). Finally, in the testis of some fish species (*Larimichthys polyactis* and *Boleophthalmus pectinirostris*), kinesin-2 (specifically KIF3A) is highly expressed and colocalizes with mitochondria in the nuclear periphery during spermiogenesis and in the midpiece of mature sperm (Wang et al., 2019; Zhao et al., 2018), suggesting an active role in mitochondrial positioning during sperm maturation.

### Dividing cells

During cell division, mitochondria are divided between the two daughter cells by either active or passive mechanisms. In metaphase, phosphorylation triggers the release of the dynein and kinesin motors from the Miro–TRAK adaptor complex on the mitochondrial surface, causing mitochondria to dissociate from MTs, ensuring symmetrical mitochondrial distribution and inheritance (Chung et al., 2016). Thus, passive inheritance results from the active release of motor proteins from mitochondria. In contrast, at later stages of mitosis, both MT- and actin-based motors play an active role in mitochondrial segregation between the two daughter cells. During anaphase, MYO19 has been shown to tether mitochondria to actin filaments, thereby ensuring equal distribution between the two daughter cells (Rohn et al., 2014). In cells undergoing cytokinesis, mitochondria are linked via kinesin-1 to the growing tips of astral MTs of the mitotic spindle and thereby are transported to the cleavage furrow (Lawrence and Mandato, 2013a; Lawrence et al., 2016; Lawrence and Mandato, 2013b). In addition, Miro1/2-dependent recruitment of the centromeric protein F (CENP-F), a non-motor MT-binding protein that associates with growing MTs, to mitochondria is required for their transport towards the periphery of the two daughter cells in late cytokinesis (Kanfer et al., 2015, 2017). All these processes are likely to involve Miro1/2, which coordinates mitochondrial distribution by MT- and actin-based motors, since Miro1/2 double-KO MEFs display aberrant mitochondrial positioning resulting from asymmetric mitochondrial segregation during mitosis (López-Domenéch et al., 2018).

## Mitochondrial dynamics: fission and fusion

Mitochondria undergo regulated fission and fusion events to maintain a healthy, dynamic network (Giacomello et al., 2020; Tilokani et al., 2018). In mammals, the major mediators of fission and fusion are members of the dynamin-related GTPase family and include mitofusins 1 and 2 (MFN1 and MFN2, respectively), optic atrophy 1 (OPA1) and dynamin-related protein 1 (DRP1, also known as DNM1L).

### Fission

Mitochondrial fission first involves separation of the IMM followed by OMM division (Chakrabarti et al., 2018; Cho et al., 2017; Labrousse et al., 1999; Lee and Yoon, 2014; Legesse-Miller et al., 2003). Endoplasmic reticulum (ER) tubules wrap around mitochondria, inducing a first constriction at these ER–mitochondria contacts, which mark the sites of mitochondrial fission as well as of active mtDNA replication (Friedman et al., 2011; Korobova et al., 2013; Lewis et al., 2016).

**Fig. 2.**
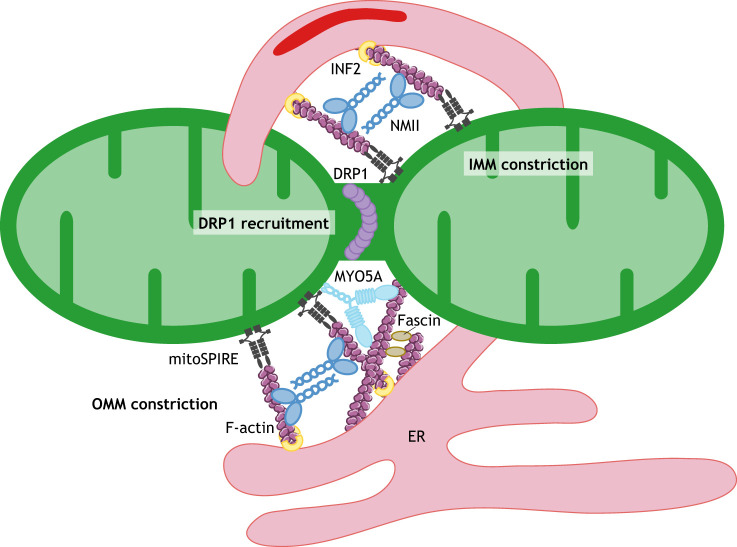
**Motor proteins and cytoskeletal machinery regulating mitochondrial fission.** Under steady-state conditions, the dynamic assembly of actin filaments onto mitochondria occurs at ER–mitochondria contact sites. Here, increased mitochondrial matrix Ca^2+^ activates the ER-bound formin INF2, resulting in actin polymerization. NMIIA provides the force to draw the ER and mitochondria closer together. At these ER–mitochondria contact sites, Ca^2+^ is transferred from the ER to mitochondria, triggering IMM constriction through an as-yet-unknown mechanism. Recruitment of the fission factor DRP1 from the cytosol to mitochondria involves several motor proteins (not shown). INF2 cooperates with mitoSPIRE and the actin bundling-protein fascin to drive polymerization of actin filaments on the mitochondrial surface, which target DRP1 to mitochondrial fission sites and stimulate its oligomerization. MYO5A, which interacts with mitoSPIRE, is recruited adjacent to DRP1 on mitochondria and facilitates fission; however, the exact role of MYO5A and actin filaments in this process remains to be established. Finally, NMII accumulates at mitochondrial constriction sites enabling DRP1 oligomerization and, by pulling actin filaments, provides the force for mitochondrial constriction. Actin filament images are adapted from Servier Medical Art (https://smart.servier.com/) under the terms of a CC-BY 3.0 license.

The machinery driving IMM fission in the matrix is poorly understood; however, the process is triggered by a transient rise in mitochondrial matrix Ca^2+^ that occurs at ER–mitochondria contact sites, which are generated and stabilized by the actin cytoskeleton (Cho et al., 2017). The formin INF2, an actin nucleator embedded in the ER membrane through a prenylated C-terminal CAAX motif, polymerizes actin away from the ER (Fig. 2) (Chhabra et al., 2009). These actin filaments together with NMIIA (which has a heavy chain encoded by *MYH9*), which forms bipolar mini-filaments, are thought to provide the contractile tension to draw the ER and mitochondria closer together, thereby stimulating ER-to-mitochondrial Ca^2+^ transfer (Chakrabarti et al., 2018).

OMM scission requires the dynamic recruitment of DRP1 from the cytosol to tail-anchored proteins that act as DRP1 receptors (Fig. 2), such as the mitochondrial fission factor (MFF) and the mitochondria dynamics proteins 49 and 51 (MiD49 and MiD51, also known as MIEF2 and MIEF1, respectively) (Giacomello et al., 2020). DRP1 oligomerizes into ring-like structures around mitochondria, causing initial constriction of the organelle, before GTP hydrolysis leads to further mitochondrial membrane constriction (Tilokani et al., 2018). The final scission event downstream of DRP1 has recently been proposed to involve Golgi-derived vesicles (Fonseca et al., 2019; Nagashima et al., 2020) and occur at mitochondria–lysosome contact sites (Wong et al., 2018). In addition, several MT-based motors regulate DRP1 recruitment from the cytosol to mitochondria. Kinesin-3 (specifically the KIF1Bβ isoform) drives mitochondrial fission by activating calcineurin, which dephosphorylates DRP1 at Ser^637^, leading to DRP1 translocation to mitochondria (Li et al., 2016). In contrast, under oxidative stress, kinesin-1 has been shown to enhance DRP1 phosphorylation at Ser^616^ through JNK activation, which also promotes DRP1 recruitment to mitochondria (Perdiz et al., 2017; Reed et al., 2006). Finally, the dynein–dynactin complex also facilitates DRP1 translocation to mitochondria, since disruption of dynein function by overexpression of the dynactin subunit p50 (also known as DCTN2) in HeLa cells leads to accumulation of DRP1 in the cytosol, resulting in the formation of long, highly branched mitochondria (Varadi et al., 2004). Interestingly, in ageing *Dync1h1*-mutant mice, mitochondria progressively increase in size and become profoundly dysfunctional in muscle (Eschbach et al., 2013). In contrast to MT-based motors, Miro1/2 negatively regulates DRP1 recruitment to mitochondria, thereby suppressing fission, although the recruitment of fusion-promoting factors may also be affected (Covill-Cooke et al., 2020; Ding et al., 2016; Saotome et al., 2008).

OMM constriction is mediated by myosin motor proteins and the actin cytoskeleton (Fig. 2). INF2, which directly interacts and cooperates with mitoSPIRE and the actin-bundling protein fascin, drives actin polymerization on the mitochondrial surface (Korobova et al., 2013; Lin et al., 2019; Manor et al., 2015; Yang and Svitkina, 2019). These mitochondrial actin filaments not only directly bind DRP1 and target it to mitochondrial fission sites, but also stimulate DRP1 oligomerization (De Vos et al., 2005; Hatch et al., 2016; Ji et al., 2017, 2015; Korobova et al., 2013). In melanoma cell lines, mitoSPIRE binds directly to MYO5A, which recruits this motor to mitochondrial fission sites adjacent to DRP1 puncta; MYO5A recruitment facilitates mitochondrial fission, whereas MYO5A depletion leads to mitochondrial elongation (Araujo et al., 2019 preprint). NMIIA, NMIIB and NMIIC (which have heavy chains encoded by *MYH9*, *MYH10* and *MYH14*, respectively) also accumulate at mitochondrial constriction sites and enable DRP1 oligomerization, as inhibition of NMII activity leads to increased mitochondrial length and loss of mitochondrial DRP1 (Almutawa et al., 2019; DuBoff et al., 2012; Hatch et al., 2014; Ji et al., 2015; Korobova et al., 2013; Yang and Svitkina, 2019). Furthermore, bipolar NMII filaments can pull on actin filaments to provide the tension force for mitochondrial constriction (Friedman et al., 2011; Osellame et al., 2016). Interestingly, NMIIC with the R94L heavy-chain mutation, which has been linked to peripheral neuropathies, acts as a dominant-negative protein that inhibits fission (Almutawa et al., 2019). In cardiomyocytes after ischaemia–reperfusion injury, inhibition of myosin II with blebbistatin or loss of NMIIA reduces mitochondrial fission and mitophagy (Li et al., 2018).

Finally, dynamic assembly and disassembly of actin filaments onto mitochondrial subpopulations has been observed at ER–mitochondria contact sites; this is blocked by inhibition of actin nucleators, such as the ARP2/3 complex and formins (Moore et al., 2016). This cycling is suggested to act as a steady-state surveillance method to modulate the mitochondrial fission–fusion balance to promote network remodelling and content mixing (Fung et al., 2019; Moore et al., 2016).

### Fusion

OMM fusion requires tethering of MFN1 and MFN2 on the OMM between adjacent mitochondria followed by GTP hydrolysis (Tilokani et al., 2018). IMM fusion is mediated by OPA1, which is inserted into the IMM and is cleaved at S1 and S2 sites by two membrane-bound metalloproteases, OMA1 and YME1L1, resulting in at least five OPA1 fragments (two larger L-OPA1 fragments and three shorter S-OPA1 fragments) thought to regulate fusion (Giacomello et al., 2020).

The importance of the cytoskeleton for mitochondrial fusion is less well understood. Inhibiting actin filament polymerization or inducing changes in MT dynamics has little effect on fusion kinetics (Mattenberger et al., 2003). Recent results, however, highlight a potential role of kinesin-3 in IMM fusion, as it binds directly to YME1L1 and its overexpression promotes cleavage of L-OPA1, which leads to mitochondrial fission and fragmentation triggering nerve growth factor (NGF)-induced apoptosis (Ando et al., 2019). However, where binding between kinesin-3 and YME1L1 occurs remains to be established, since kinesin-3 is a cytosolic protein, whereas YME1L1 is present at the IMM.

With their many links to cytoskeletal motors, Miro proteins appear to be important for maintaining the balance between fission and fusion, which controls mitochondrial morphology; however, whether this is a direct effect or due to an indirect effect on transport requires further investigation. Miro proteins may facilitate fusion, since the overexpression of the wild-type protein, or expression of a constitutively active form of the protein, results in mitochondrial enlargement in *Drosophila* and induces the formation of long thread-like mitochondria (Babic et al., 2015; Ding et al., 2016; Fransson et al., 2006; López-Domenéch et al., 2018; Saotome et al., 2008), whereas in yeast, loss of the Miro homologue Gem1 or its GTPase activity leads to fragmented mitochondria and a collapsed network (Frederick et al., 2004). Miro1/2 and Milton interact with both MFN1 and MFN2, which are also required for mitochondrial transport (Misko et al., 2010). Thus, there is crosstalk and coordination between mitochondrial transport and fusion; however, the dependence of mitochondrial fusion on transport has made it difficult to determine the precise role of Miro proteins and MFNs in mitochondrial fusion. Depletion or mutation of MFN2 reduces the rate of anterograde and retrograde mitochondrial transport in neurons (Misko et al., 2010), while reducing mitochondrial motility by suppression of Miro1/2 inhibits the rate of mitochondrial fusion (Cagalinec et al., 2013).

## Mitochondrial turnover and quality control

Several pathways are known to regulate the turnover of whole mitochondria by mitophagy; one of the best studied pathways involves the serine/threonine kinase PINK1 and the E3 ubiquitin ligase Parkin (PRKN), which when mutated cause autosomal recessive Parkinson's disease (Narendra et al., 2008; Ryan et al., 2015). A hallmark of mitochondrial damage is membrane depolarization, leading to the accumulation of PINK1 on the OMM and phosphorylation of ubiquitin attached to OMM proteins (Harper et al., 2018). This, in turn, triggers the recruitment of auto-inhibited PRKN to the OMM, where it binds to phosphorylated ubiquitin. PINK1-dependent phosphorylation leads to full PRKN activation, which then allows PRKN to ubiquitylate OMM proteins including Miro1/2, thereby amplifying the mitophagy signal. Cargo-selective autophagy receptors, such as optineurin (OPTN), Ca^2+^-binding and coiled-coil domain-containing protein 2 (CALCOCO2, also known as NDP52), and Tax1-binding protein 1 (TAX1BP1), recognize and capture ubiquitylated mitochondria through their ubiquitin-binding domains and simultaneously bind to LC3 proteins (also known as MAP1LC3) to recruit autophagosomal membranes for the formation of mitophagosomes, which ultimately fuse with lysosomes for degradation (Pickles et al., 2018).

**Fig. 3.**
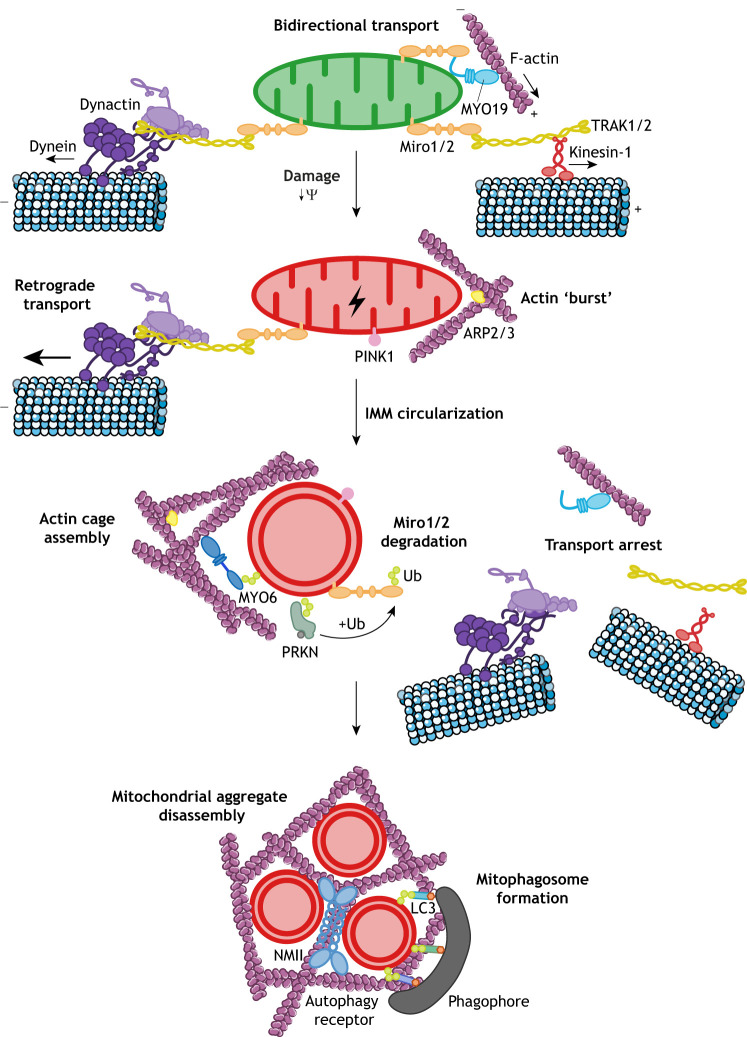
**Motor proteins and machinery associated with damaged mitochondria during mitophagy.** Healthy mitochondria are actively transported by kinesin-1 and dynein motors and positioned by MYO19. Upon mitochondrial damage, loss of mitochondrial membrane potential (Ψ) results in the accumulation of the kinase PINK1 on the OMM. Within 5 min of damage induction, a short ‘burst’ of mitochondrial actin can be observed, which requires the ARP2/3 complex and leads to IMM circularization followed by actin disassembly. The ubiquitin ligase PRKN translocates from the cytosol to the mitochondria where it ubiquitylates (+Ub) Miro proteins, leading to their subsequent proteasomal degradation. This results in release of kinesin-1, dynein–dynactin and MYO19 from mitochondria and the arrest of mitochondrial transport. At ∼2 h after mitochondrial damage, the OMM has fragmented and MYO6 is recruited to PRKN-mediated ubiquitin chains on the mitochondrial surface, where it assembles an actin cage to isolate damaged, fragmented mitochondria, thereby preventing their re-fusion with neighbouring populations. The actin meshwork around mitochondria, together with NMII, may reorganize to disrupt any mitochondrial aggregates, creating small ‘bite-size’ mitochondria, and serve as a structural scaffold for the growing LC3-positive phagophore. Actin filament and microtubule images are adapted from Servier Medical Art (https://smart.servier.com/) under the terms of a CC-BY 3.0 license.

Before damaged mitochondria are eliminated by mitophagy, they are transported by dynein towards the cell body/soma (Fig. 3) (Cai et al., 2012; Miller and Sheetz, 2004), and defects in retrograde transport of senescent mitochondria lead to increased autophagy in axonal swellings (Pilling et al., 2006). Enhancing retrograde transport by inducing binding of mitochondria to dynein increases neuronal mitophagy (Zheng et al., 2019), whereas *Dync1h1* mutations lead to impaired perinuclear clustering of damaged mitochondria in fibroblasts (Eschbach et al., 2013). Furthermore, the release of the MT anchor SNPH from stressed mitochondria enhances retrograde mitochondrial transport before activation of PRKN-mediated mitophagy, whereas overexpression of SNPH blocks mitophagy (Lin et al., 2017). Compartmental restriction of mitophagy to the soma of stressed neurons (Evans and Holzbaur, 2020) is supported by *in vivo* data from *Drosophila* neurons (Devireddy et al., 2015; Sung et al., 2016) and Purkinje cells from *mito*-QC mice expressing a pH-sensitive fluorescent mitochondrial marker (McWilliams et al., 2016); however, clearance of damaged mitochondria has also been shown to occur locally in distal axons in response to acute stress (Ashrafi et al., 2014).

In addition to phosphorylating PRKN and ubiquitin, PINK1 also phosphorylates Miro1/2, thereby facilitating PRKN recruitment to Miro proteins (Shlevkov et al., 2016; Wang et al., 2011). Ubiquitylation of Miro1/2 by PRKN then leads to its subsequent proteasomal degradation (Fig. 3) (Birsa et al., 2014). This results in the release of MYO19 and kinesin from the mitochondrial surface, leading to a reduced motility of damaged mitochondria (Liu et al., 2012; López-Domenéch et al., 2018; Safiulina et al., 2019; Shlevkov et al., 2016; Tsai et al., 2014; Wang et al., 2011; Weihofen et al., 2009). Hence, arresting mitochondrial transport is an early step in the clearance of dysfunctional mitochondria, which potentially isolates them before their elimination by mitophagy (Fig. 3).

Mitochondrial damage not only halts the retrograde dynein-dependent movement of mitochondria, but also leads to distinct F-actin assembly events on mitochondria that are linked to recovery, segregation and the initiation of downstream mitophagy. A rapid, dynamic and transient ‘burst’ in mitochondria-localized F-actin occurs within minutes (∼5 min) of damage and requires ARP2/3 (Fig. 3), but is independent of INF2 and MYO6 (Fung et al., 2019; Kruppa et al., 2018; Li et al., 2015). This leads to circularization of mitochondria, which is their last chance to recover before being committed to clearance by mitophagy. Prolonged depolarization leads to mitochondrial fragmentation and initiates a second wave of MYO6-dependent actin polymerization after several hours, which initiates the formation of actin cages around damaged mitochondria during PRKN-mediated mitophagy (Fu and Lippincott-Schwartz, 2018; Kruppa et al., 2018). MYO6 forms a complex with PRKN and is selectively recruited to damaged, ubiquitylated mitochondria (Kruppa et al., 2018; Sarraf et al., 2013). Signalling downstream of the Rho family GTPase CDC42, as well as actin nucleators, such as the ARP2/3 complex, formins and N-WASP (also known as WASL), are all required for actin cage assembly. These MYO6-induced actin cages serve as a quality control mechanism at the onset of mitophagy by isolating dysfunctional mitochondria, thereby preventing their re-fusion with neighbouring populations and reintegration into the network (Hsieh and Yang, 2019; Kruppa et al., 2018) (Fig. 3).

In recent years, there has been growing evidence that the cytoskeletal machinery plays crucial roles during mitophagy; therefore, the actin filaments assembled by MYO6 on the mitochondrial surface may not only form actin cages, but also have important roles during mitophagy by facilitating phagophore formation and mitophagosome maturation (Kast and Dominguez, 2017; Kruppa and Buss, 2018; Kruppa et al., 2016). Indeed, since MYO6-dependent actin cages restrict the size of mitochondrial fragments, they may create small, ‘bite-size’ mitochondria that are more easily engulfed by the autophagy machinery. The synchronous formation of mitochondrial actin structures near autophagy initiation sites suggests a coordination between a reduction in mitochondrial dimensions and mitophagosome formation (Hsieh and Yang, 2019). This is driven by NMII and F-actin (Fig. 3), which have been implicated in disassembling mitochondrial aggregates into smaller pieces (1.5 h after damage) in preparation for efficient mitophagy into autophagosomes (with a typical diameter of 0.5–1.5 µm) (Hsieh and Yang, 2019; Mizushima et al., 2002).

Furthermore, the actin meshwork around mitochondria may persist after the initial damage and therefore could serve as a structural scaffold for the growing phagophore (Fig. 3) as well as supporting fusion of individual phagophores into a single mitophagosome (Hsieh and Yang, 2019; Kruppa and Buss, 2018; Tang et al., 2011). Mitochondrial actin may also drive autophagosome generation by disrupting mitochondrial aggregates to increase the available surface accessible to phagophores (Hsieh and Yang, 2019). Indeed, blocking the disassembly of mitochondrial aggregates and actin generation with an ARP2/3 inhibitor leads to prolonged clustering and inhibited mitophagosome formation (Hsieh and Yang, 2019).

## Conclusions and perspectives

In this Review, we have highlighted a distinct subset of MT- and actin-based motor proteins that are linked to mitochondrial transport, fission, fusion and turnover. Even though we have discussed these key mitochondrial processes in separate sections, they are of course intimately linked and interdependent, and thus studying the precise roles of motors and their adaptors in isolation has been difficult due to their overlapping functions. One important avenue for future research is the regulation of motors and their adaptor proteins on mitochondria in response to changes in cell physiology and metabolism. The balance between fission and fusion, as well as transport and anchoring, are fine-tuned and require the coordination and handover of MT- and actin-based motors. Furthermore, a detailed understanding of motor protein regulation in maintaining mitochondrial homeostasis is crucial to elucidate how defects in these pathways can lead to human diseases.

## Supplementary Material

Supplementary information
