# Supplementary material for: Motor proteins at the mitochondria–cytoskeleton interface
Source: J Cell Sci. 2021 Apr 15;134(7):jcs226084. doi: 10.1242/jcs.226084 (PMC8077471; doi:10.1242/jcs.226084)
Supplement: Supplementary information [file joces-134-226084-s1.pdf]

Table S1: Mutations in mitochondria-linked motor proteins and adaptors causing human diseases

Gene	Disease	Reference
MYH9 (NMMIIA)	Autosomal dominant non-syndromic deafness 17 (DFNA17)	(Dantas et al., 2014; Hildebrand et al., 2006; Lalwani et al., 2000)
	Macrothrombocytopenia and granulocyte inclusions with or without nephritis or sensorineural hearing loss (MATINS)	(Balduini et al., 2011; Heath et al., 2001; Kelley et al., 2000; Kunishima et al., 2001a; Kunishima et al., 2001b; Pecci et al., 2005; Seri et al., 2003)
MYH14 (NMMIIC)	Autosomal dominant non-syndromic deafness 4A (DFNA4A)	(Donaudy et al., 2004)
	Peripheral neuropathy, myopathy, hoarseness and hearing loss (PNMHH)	(Almutawa et al., 2019; Choi et al., 2011)
MYO5A	Griscelli syndrome type 1	(Menasche et al., 2002; Pastural et al., 1997; Pastural et al., 2000)
MYO6	Autosomal dominant deafness 22 (DFAD22)	(Hilgert et al., 2008; Melchionda et al., 2001; Sanggaard et al., 2008)
	Autosomal recessive deafness 37 (DFAR37)	(Ahmed et al., 2003)
	Autosomal dominant deafness with hypertrophic cardiomyopathy (DFNHCM)	(Mohiddin et al., 2004)
KIF1B (Kinesin-3)	Charcot-Marie-Tooth disease type 2A1 (CMT2A1)	(Drew et al., 2015; Zhao et al., 2001)
	Neuroblastoma 1 (NBLST1) Pheochromocytoma (PCC)	(Schlisio et al., 2008)
KBP (KIFBP)	Goldberg-Shprintzen megacolon syndrome (GOSHS)	(Brooks et al., 2005; Drevillon et al., 2013; Valence et al., 2013)
KIF5A (Kinesin-1)	Amyotrophic lateral sclerosis type 25 (ALS25)	(Brenner et al., 2018; Nicolas et al., 2018)
	Hereditary Spastic Paraplegia (SPG10)	(Crimella et al., 2012; Goizet et al., 2009; Reid et al., 2002)
	Neonatal intractable myoclonus (NEIMY)	(DaRe et al., 2013; Duis et al., 2016; Rydzanicz et al., 2017)
KIF5C (Kinesin-1)	Cortical dysplasia complex with other brain malformations 2 (CDCBM2)	(de Ligt et al., 2012; Poirier et al., 2013)
DYNC1H1 (Dynein, DHC)	Charcot-Marie-Tooth disease type 20 (CMT20, axonal)	(Weedon et al., 2011)
	Mental retardation autosomal dominant 13 (MRD13)	(Jamuar et al., 2014; Poirier et al., 2013; Vissers et al., 2010; Willemsen et al., 2012)
	Spinal muscular atrophy, lower extremity-predominant 1, autosomal dominant (SMA-LED1)	(Harms et al., 2010; Harms et al., 2012; Tsurusaki et al., 2012)
DYNC1I2 (Dynein, DIC)	Neurodevelopmental disorder with microcephaly and structural brain anomalities	(Ansar et al., 2019)
DCTN1 (Dynactin, p150^{Glued})	Distal hereditary motor neuronopathy 7B (HMN7B)	(Puls et al., 2003)
	Amyotrophic lateral sclerosis	(Munch et al., 2004)
	Perry syndrome (PERRYS)	(Farrer et al., 2009)
TRAK1	Epileptic encephalopathy, early infantile 68 (EIEE68)	(Anazi et al., 2017; Barel et al., 2017; Sagie et al., 2018)

Supplementary References

Ahmed, Z. M., Morell, R. J., Riazuddin, S., Gropman, A., Shaukat, S., Ahmad, M. M., Mohiddin, S. A., Fananapazir, L., Caruso, R. C., Husnain, T. et al. (2003). Mutations of MYO6 are associated with recessive deafness, DFNB37. *Am J Hum Genet* **72**, 1315-22.

Almutawa, W., Smith, C., Sabouny, R., Smit, R. B., Zhao, T., Wong, R., Lee-Glover, L., Desrochers-Goyette, J., Ilamathi, H. S., Care4Rare Canada, C. et al. (2019). The R941L mutation in MYH14 disrupts mitochondrial fission and associates with peripheral neuropathy. *EBioMedicine* **45**, 379-392.

Anazi, S., Maddirevula, S., Salpietro, V., Asi, Y. T., Alsahli, S., Alhashem, A., Shamseldin, H. E., AlZahrani, F., Patel, N., Ibrahim, N. et al. (2017). Expanding the genetic heterogeneity of intellectual disability. *Hum Genet* **136**, 1419-1429.

Ansar, M., Ullah, F., Paracha, S. A., Adams, D. J., Lai, A., Pais, L., Iwaszkiewicz, J., Millan, F., Sarwar, M. T., Agha, Z. et al. (2019). Bi-allelic Variants in DYNC1I2 Cause Syndromic Microcephaly with Intellectual Disability, Cerebral Malformations, and Dysmorphic Facial Features. *Am J Hum Genet* **104**, 1073-1087.

Balduini, C. L., Pecci, A. and Savoia, A. (2011). Recent advances in the understanding and management of MYH9-related inherited thrombocytopenias. *Br J Haematol* **154**, 161-74.

Barel, O., Malicdan, M. C. V., Ben-Zeev, B., Kandel, J., Pri-Chen, H., Stephen, J., Castro, I. G., Metz, J., Atawa, O., Moshkovitz, S. et al. (2017). Deleterious variants in TRAK1 disrupt mitochondrial movement and cause fatal encephalopathy. *Brain* **140**, 568-581.

Brenner, D., Yilmaz, R., Muller, K., Grehl, T., Petri, S., Meyer, T., Grosskreutz, J., Weydt, P., Ruf, W., Neuwirth, C. et al. (2018). Hot-spot KIF5A mutations cause familial ALS. *Brain* **141**, 688-697.

Brooks, A. S., Bertoli-Avella, A. M., Burzynski, G. M., Breedveld, G. J., Osinga, J., Boven, L. G., Hurst, J. A., Mancini, G. M., Lequin, M. H., de Coo, R. F. et al. (2005). Homozygous nonsense mutations in KIAA1279 are associated with malformations of the central and enteric nervous systems. *Am J Hum Genet* **77**, 120-6.

Choi, B. O., Kang, S. H., Hyun, Y. S., Kanwal, S., Park, S. W., Koo, H., Kim, S. B., Choi, Y. C., Yoo, J. H., Kim, J. W. et al. (2011). A complex phenotype of peripheral neuropathy, myopathy, hoarseness, and hearing loss is linked to an autosomal dominant mutation in MYH14. *Hum Mutat* **32**, 669-77.

Crimella, C., Baschirotto, C., Arnoldi, A., Tonelli, A., Tenderini, E., Airoldi, G., Martinuzzi, A., Trabacca, A., Losito, L., Scarlato, M. et al. (2012). Mutations in the motor and stalk domains of KIF5A in spastic paraplegia type 10 and in axonal Charcot-Marie-Tooth type 2. *Clin Genet* **82**, 157-64.

Dantas, V. G., Lezirovitz, K., Yamamoto, G. L., Moura de Souza, C. F., Ferreira, S. G. and Mingroni-Netto, R. C. (2014). c.G2114A MYH9 mutation (DFNA17) causes non-syndromic autosomal dominant hearing loss in a Brazilian family. *Genet Mol Biol* **37**, 616-21.

DaRe, J. T., Vasta, V., Penn, J., Tran, N. T. and Hahn, S. H. (2013). Targeted exome sequencing for mitochondrial disorders reveals high genetic heterogeneity. *BMC Med Genet* **14**, 118.

de Ligt, J., Willemsen, M. H., van Bon, B. W., Kleefstra, T., Yntema, H. G., Kroes, T., Vulto-van Silfhout, A. T., Koolen, D. A., de Vries, P., Gilissen, C. et al. (2012). Diagnostic exome sequencing in persons with severe intellectual disability. *N Engl J Med* **367**, 1921-9.

Donaudy, F., Snoeckx, R., Pfister, M., Zenner, H. P., Blin, N., Di Stazio, M., Ferrara, A., Lanzara, C., Ficarella, R., Declau, F. et al. (2004). Nonmuscle myosin heavy-chain gene MYH14 is

expressed in cochlea and mutated in patients affected by autosomal dominant hearing impairment (DFNA4). *Am J Hum Genet* **74**, 770-6.

Drevillon, L., Megarbane, A., Demeer, B., Matar, C., Benit, P., Briand-Suleau, A., Bodereau, V., Ghoumid, J., Nasser, M., Decrouy, X. et al. (2013). KBP-cytoskeleton interactions underlie developmental anomalies in Goldberg-Shprintzen syndrome. *Hum Mol Genet* **22**, 2387-99.

Drew, A. P., Zhu, D., Kidambi, A., Ly, C., Tey, S., Brewer, M. H., Ahmad-Annuar, A., Nicholson, G. A. and Kennerson, M. L. (2015). Improved inherited peripheral neuropathy genetic diagnosis by whole-exome sequencing. *Mol Genet Genomic Med* **3**, 143-54.

Duis, J., Dean, S., Applegate, C., Harper, A., Xiao, R., He, W., Dollar, J. D., Sun, L. R., Waberski, M. B., Crawford, T. O. et al. (2016). KIF5A mutations cause an infantile onset phenotype including severe myoclonus with evidence of mitochondrial dysfunction. *Ann Neurol* **80**, 633-7.

Farrer, M. J., Hulihan, M. M., Kachergus, J. M., Dachsel, J. C., Stoessl, A. J., Grantier, L. L., Calne, S., Calne, D. B., Lechevalier, B., Chapon, F. et al. (2009). DCTN1 mutations in Perry syndrome. *Nat Genet* **41**, 163-5.

Goizet, C., Boukhris, A., Mundwiller, E., Tallaksen, C., Forlani, S., Toutain, A., Carriere, N., Paquis, V., Depienne, C., Durr, A. et al. (2009). Complicated forms of autosomal dominant hereditary spastic paraplegia are frequent in SPG10. *Hum Mutat* **30**, E376-85.

Harms, M. B., Allred, P., Gardner, R., Jr., Fernandes Filho, J. A., Florence, J., Pestronk, A., Al-Lozi, M. and Baloh, R. H. (2010). Dominant spinal muscular atrophy with lower extremity predominance: linkage to 14q32. *Neurology* **75**, 539-46.

Harms, M. B., Ori-McKenney, K. M., Scoto, M., Tuck, E. P., Bell, S., Ma, D., Masi, S., Allred, P., Al-Lozi, M., Reilly, M. M. et al. (2012). Mutations in the tail domain of DYNC1H1 cause dominant spinal muscular atrophy. *Neurology* **78**, 1714-20.

Heath, K. E., Campos-Barros, A., Toren, A., Rozenfeld-Granot, G., Carlsson, L. E., Savige, J., Denison, J. C., Gregory, M. C., White, J. G., Barker, D. F. et al. (2001). Nonmuscle myosin heavy chain IIA mutations define a spectrum of autosomal dominant macrothrombocytopenias: May-Hegglin anomaly and Fechtner, Sebastian, Epstein, and Alport-like syndromes. *Am J Hum Genet* **69**, 1033-45.

Hildebrand, M. S., de Silva, M. G., Gardner, R. J., Rose, E., de Graaf, C. A., Bahlo, M. and Dahl, H. H. (2006). Cochlear implants for DFNA17 deafness. *Laryngoscope* **116**, 2211-5.

Hilgert, N., Topsakal, V., van Dinther, J., Offeciers, E., Van de Heyning, P. and Van Camp, G. (2008). A splice-site mutation and overexpression of MYO6 cause a similar phenotype in two families with autosomal dominant hearing loss. *Eur J Hum Genet* **16**, 593-602.

Jamuar, S. S., Lam, A. T., Kircher, M., D'Gama, A. M., Wang, J., Barry, B. J., Zhang, X., Hill, R. S., Partlow, J. N., Rozzo, A. et al. (2014). Somatic mutations in cerebral cortical malformations. *N Engl J Med* **371**, 733-43.

Kelley, M. J., Jawien, W., Ortel, T. L. and Korczak, J. F. (2000). Mutation of MYH9, encoding non-muscle myosin heavy chain A, in May-Hegglin anomaly. *Nat Genet* **26**, 106-8.

Kunishima, S., Kojima, T., Matsushita, T., Tanaka, T., Tsurusawa, M., Furukawa, Y., Nakamura, Y., Okamura, T., Amemiya, N., Nakayama, T. et al. (2001a). Mutations in the NMMHC-A gene cause autosomal dominant macrothrombocytopenia with leukocyte inclusions (May-Hegglin anomaly/Sebastian syndrome). *Blood* **97**, 1147-9.

Kunishima, S., Matsushita, T., Kojima, T., Amemiya, N., Choi, Y. M., Hosaka, N., Inoue, M., Jung, Y., Mamiya, S., Matsumoto, K. et al. (2001b). Identification of six novel MYH9 mutations and genotype-phenotype relationships in autosomal dominant macrothrombocytopenia with leukocyte inclusions. *J Hum Genet* **46**, 722-9.

Journal of Cell Science • Supplementary information

Lalwani, A. K., Goldstein, J. A., Kelley, M. J., Luxford, W., Castelein, C. M. and Mhatre, A. N. (2000). Human Nonsyndromic Hereditary Deafness DFNA17 Is Due to a Mutation in Nonmuscle Myosin MYH9. *The American Journal of Human Genetics* **67**, 1121-1128.

Melchionda, S., Ahituv, N., Bisceglia, L., Sobe, T., Glaser, F., Rabionet, R., Arbones, M. L., Notarangelo, A., Di Iorio, E., Carella, M. et al. (2001). MYO6, the human homologue of the gene responsible for deafness in Snell's waltzer mice, is mutated in autosomal dominant nonsyndromic hearing loss. *Am J Hum Genet* **69**, 635-40.

Menasche, G., Fischer, A. and de Saint Basile, G. (2002). Griscelli syndrome types 1 and 2. *Am J Hum Genet* **71**, 1237-8; author reply 1238.

Mohiddin, S. A., Ahmed, Z. M., Griffith, A. J., Tripodi, D., Friedman, T. B., Fananapazir, L. and Morell, R. J. (2004). Novel association of hypertrophic cardiomyopathy, sensorineural deafness, and a mutation in unconventional myosin VI (MYO6). *J Med Genet* **41**, 309-14.

Munch, C., Sedlmeier, R., Meyer, T., Homberg, V., Sperfeld, A. D., Kurt, A., Prudlo, J., Peraus, G., Hanemann, C. O., Stumm, G. et al. (2004). Point mutations of the p150 subunit of dynactin (DCTN1) gene in ALS. *Neurology* **63**, 724-6.

Nicolas, A. Kenna, K. P. Renton, A. E. Ticozzi, N. Faghri, F. Chia, R. Dominov, J. A. Kenna, B. J. Nalls, M. A. Keagle, P. et al. (2018). Genome-wide Analyses Identify KIF5A as a Novel ALS Gene. *Neuron* **97**, 1268-1283 e6.

Pastural, E., Barrat, F. J., Dufourcq-Lagelouse, R., Certain, S., Sanal, O., Jabado, N., Seger, R., Griscelli, C., Fischer, A. and de Saint Basile, G. (1997). Griscelli disease maps to chromosome 15q21 and is associated with mutations in the myosin-Va gene. *Nat Genet* **16**, 289-92.

Pastural, E., Ersoy, F., Yalman, N., Wulffraat, N., Grillo, E., Ozkinay, F., Tezcan, I., Gedikoglu, G., Philippe, N., Fischer, A. et al. (2000). Two genes are responsible for Griscelli syndrome at the same 15q21 locus. *Genomics* **63**, 299-306.

Pecci, A., Canobbio, I., Balduini, A., Stefanini, L., Cisterna, B., Marseglia, C., Noris, P., Savoia, A., Balduini, C. L. and Torti, M. (2005). Pathogenetic mechanisms of hematological abnormalities of patients with MYH9 mutations. *Hum Mol Genet* **14**, 3169-78.

Poirier, K., Lebrun, N., Broix, L., Tian, G., Saillour, Y., Boscheron, C., Parrini, E., Valence, S., Pierre, B. S., Oger, M. et al. (2013). Mutations in TUBG1, DYNC1H1, KIF5C and KIF2A cause malformations of cortical development and microcephaly. *Nat Genet* **45**, 639-47.

Puls, I., Jonnakuty, C., LaMonte, B. H., Holzbaur, E. L., Tokito, M., Mann, E., Floeter, M. K., Bidus, K., Drayna, D., Oh, S. J. et al. (2003). Mutant dynactin in motor neuron disease. *Nat Genet* **33**, 455-6.

Reid, E., Kloos, M., Ashley-Koch, A., Hughes, L., Bevan, S., Svenson, I. K., Graham, F. L., Gaskell, P. C., Dearlove, A., Pericak-Vance, M. A. et al. (2002). A kinesin heavy chain (KIF5A) mutation in hereditary spastic paraplegia (SPG10). *Am J Hum Genet* **71**, 1189-94.

Rydzanicz, M., Jagla, M., Kosinska, J., Tomasik, T., Sobczak, A., Pollak, A., Herman-Sucharska, I., Walczak, A., Kwinta, P. and Ploski, R. (2017). KIF5A de novo mutation associated with myoclonic seizures and neonatal onset progressive leukoencephalopathy. *Clin Genet* **91**, 769-773.

Sagie, S., Lerman-Sagie, T., Maljevic, S., Yosovich, K., Detert, K., Chung, S. K., Rees, M. I., Lerche, H. and Lev, D. (2018). Expanding the phenotype of TRAK1 mutations: hyperekplexia and refractory status epilepticus. *Brain* **141**, e55.

Sanggaard, K. M., Kjaer, K. W., Eiberg, H., Nurnberg, G., Nurnberg, P., Hoffman, K., Jensen, H., Sorum, C., Rendtorff, N. D. and Tranebjaerg, L. (2008). A novel nonsense mutation in

Journal of Cell Science • Supplementary information

MYO6 is associated with progressive nonsyndromic hearing loss in a Danish DFNA22 family. *Am J Med Genet A* **146A**, 1017-25.

Schlisio, S., Kenchappa, R. S., Vredeveld, L. C., George, R. E., Stewart, R., Greulich, H., Shahriari, K., Nguyen, N. V., Pigny, P., Dahia, P. L. et al. (2008). The kinesin KIF1Bbeta acts downstream from EglN3 to induce apoptosis and is a potential 1p36 tumor suppressor. *Genes Dev* **22**, 884-93.

Seri, M., Pecci, A., Di Bari, F., Cusano, R., Savino, M., Panza, E., Nigro, A., Noris, P., Gangarossa, S., Rocca, B. et al. (2003). MYH9-Related Disease: May-Hegglin Anomaly, Sebastian Syndrome, Fechtner Syndrome, and Epstein Syndrome Are not Distinct Entities but Represent a Variable Expression of a Single Illness. *Medicine* **82**.

Tsurusaki, Y., Saitoh, S., Tomizawa, K., Sudo, A., Asahina, N., Shiraishi, H., Ito, J., Tanaka, H., Doi, H., Saitsu, H. et al. (2012). A DYNC1H1 mutation causes a dominant spinal muscular atrophy with lower extremity predominance. *neurogenetics* **13**, 327-32.

Valence, S., Poirier, K., Lebrun, N., Saillour, Y., Sonigo, P., Bessieres, B., Attie-Bitach, T., Benachi, A., Masson, C., Encha-Razavi, F. et al. (2013). Homozygous truncating mutation of the KBP gene, encoding a KIF1B-binding protein, in a familial case of fetal polymicrogyria. *neurogenetics* **14**, 215-24.

Vissers, L. E., de Ligt, J., Gilissen, C., Janssen, I., Steehouwer, M., de Vries, P., van Lier, B., Arts, P., Wieskamp, N., del Rosario, M. et al. (2010). A de novo paradigm for mental retardation. *Nat Genet* **42**, 1109-12.

Weedon, M. N., Hastings, R., Caswell, R., Xie, W., Paszkiewicz, K., Antoniadi, T., Williams, M., King, C., Greenhalgh, L., Newbury-Ecob, R. et al. (2011). Exome sequencing identifies a DYNC1H1 mutation in a large pedigree with dominant axonal Charcot-Marie-Tooth disease. *Am J Hum Genet* **89**, 308-12.

Willemsen, M. H., Vissers, L. E., Willemsen, M. A., van Bon, B. W., Kroes, T., de Ligt, J., de Vries, B. B., Schoots, J., Lugtenberg, D., Hamel, B. C. et al. (2012). Mutations in DYNC1H1 cause severe intellectual disability with neuronal migration defects. *J Med Genet* **49**, 179-83.

Zhao, C., Takita, J., Tanaka, Y., Setou, M., Nakagawa, T., Takeda, S., Yang, H. W., Terada, S., Nakata, T., Takei, Y. et al. (2001). Charcot-Marie-Tooth disease type 2A caused by mutation in a microtubule motor KIF1Bbeta. *Cell* **105**, 587-97.
